# Dengue Virus Infection and Virus-Specific HLA-A2 Restricted Immune Responses in Humanized NOD-*scid IL2rγ^null^* Mice

**DOI:** 10.1371/journal.pone.0007251

**Published:** 2009-10-05

**Authors:** Smita Jaiswal, Todd Pearson, Heather Friberg, Leonard D. Shultz, Dale L. Greiner, Alan L. Rothman, Anuja Mathew

**Affiliations:** 1 Center for Infectious Disease and Vaccine Research, University of Massachusetts Medical School, Worcester, Massachusetts, United States of America; 2 Diabetes Division, University of Massachusetts Medical School, Worcester, Massachusetts, United States of America; 3 Jackson Laboratory, Bar Harbor, Maine, United States of America; New York University School of Medicine, United States of America

## Abstract

**Background:**

The lack of a suitable animal model to study viral and immunological mechanisms of human dengue disease has been a deterrent to dengue research.

**Methodology/Principal Findings:**

We sought to establish an animal model for dengue virus (DENV) infection and immunity using non-obese diabetic/severe combined immunodeficiency interleukin-2 receptor γ-chain knockout (NOD-*scid IL2rγ^null^*) mice engrafted with human hematopoietic stem cells. Human CD45^+^ cells in the bone marrow of engrafted mice were susceptible to in vitro infection using low passage clinical and established strains of DENV. Engrafted mice were infected with DENV type 2 by different routes and at multiple time points post infection, we detected DENV antigen and RNA in the sera, bone marrow, spleen and liver of infected engrafted mice. Anti-dengue IgM antibodies directed against the envelope protein of DENV peaked in the sera of mice at 1 week post infection. Human T cells that developed following engraftment of HLA-A2 transgenic NOD-*scid IL2rγ^null^* mice with HLA-A2^+^ human cord blood hematopoietic stem cells, were able to secrete IFN-γ, IL-2 and TNF-α in response to stimulation with three previously identified A2 restricted dengue peptides NS4b 2353_(111–119)_, NS4b 2423_(181–189)_, and NS4a 2148_(56–64)_.

**Conclusions/Significance:**

This is the first study to demonstrate infection of human cells and functional DENV-specific T cell responses in DENV-infected humanized mice. Overall, these mice should be a valuable tool to study the role of prior immunity on subsequent DENV infections.

## Introduction

Dengue viruses (DENV) are small enveloped RNA viruses with a single-stranded positive sense genome in the family *Flaviviridae*. Of the flaviviruses, DENV have the most significant impact on morbidity and mortality worldwide with over 100 million infections estimated annually [1]. In most cases, infection is either minimally symptomatic or results in an uncomplicated, though sometimes severe, acute febrile illness (dengue fever, DF). In a small percentage of cases, however, individuals develop a severe capillary leakage syndrome, dengue hemorrhagic fever (DHF), which can be life-threatening [2].

Studies on humans infected with DENV provide strong evidence for an immunologic basis for the pathogenesis of DHF. DHF is strongly associated with a secondary DENV infection, *i.e*. infection in individuals with pre-existing DENV-specific antibodies and memory T cells from an earlier infection with a different DENV serotype [3]–[5]. Therefore, experimental manipulation of in vivo immune responses to DENV would be a desirable approach to explore the role of prior immunity on subsequent DENV infection and to test the potential for candidate vaccines and therapeutics.

Subcutaneous injection of DENV into non-human primates results in productive infection with animals developing DENV-specific antibody and memory T cell responses [6], [7] but no clinical disease. Several murine models for DENV-induced disease have been reported that have been useful in dissecting some key aspects of disease pathogenesis [8]–[14]. However, major limitations of these models include the use of very high input doses of virus, routes of inoculation that do not mimic natural infection and the use of mouse-adapted strains of virus.

Data from several laboratories indicate that human cells represent the most susceptible cells to DENV infection. Previous attempts at engrafting immunodeficient mice with human cells as targets for DENV infection have yielded limited success partly because of low levels of human engraftment [15]. Bente and Moto et al immunized engrafted NOD-*scid* mice with clinical strains of DENV-2 [16], [17]. Infected engrafted mice developed clinical signs of dengue disease including thrombocytopenia and erythema compared to infected non-engrafted mice and uninfected engrafted mice. Kuruvilla et al used humanized BALB/c-RAG2^−/−^γ_c_^−/−^ mice (RAG hu) and demonstrated that these mice developed dengue-specific IgM and IgG antibodies several weeks after infection [18]. The data by these two groups are promising, however human T cell responses in engrafted mice were not examined in either of these studies.

Novel mouse models have recently been described that permit long term multi-lineage human hematopoiesis and immune response [19]–[21]. We utilized NOD-*scid IL2rγ^null^* mice, considered the “gold standard” strain for human hematolymphoid development to determine whether humanized mice will support productive infection with DENV [20], [22]–[24]. We identified human cells that were targets of in vitro and in vivo infection with DENV. Human T cells from infected engrafted mice produced IFN-γ, TNF-α and IL-2 in response to stimulation with three HLA A2 restricted dengue virus-specific peptides. These mice also developed IgM antibodies directed against the DENV envelope protein. Our results are a promising first step towards developing a small animal model with a functional human adaptive immune system to study human dengue virus induced immunity and disease.

## Results

### Human hematopoiesis in NOD-scid IL2rγ^null^ mice

**Figure 1:**
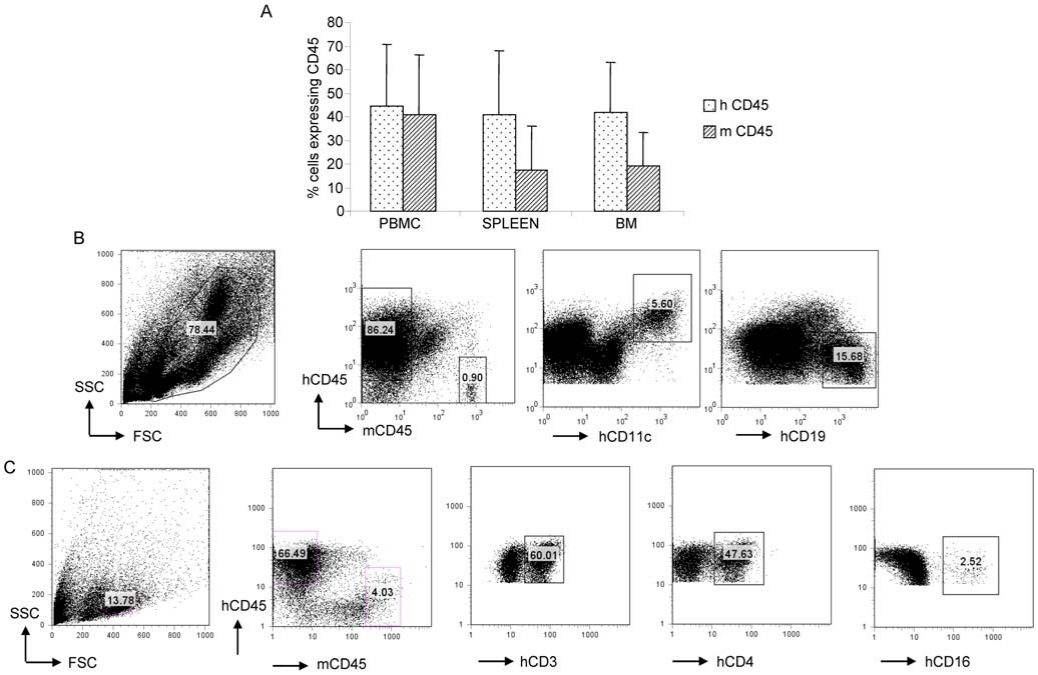
Human hematopoiesis in organs of engrafted NOD-*scid IL2rγ^null^* mice. (A) Approximately 3 months after engraftment of NOD-*scid IL2rγ^null^* mice with human T cell-depleted umbilical cord blood cells, reconstitution was assessed in target organs using flow cytometry. Values on the Y-axis represent frequencies of hCD45^+^ and mCD45^+^ cells within the mononuclear cell gates in the PBMC (n = 26), spleen (n = 31) and all cells in the bone marrow (n = 31). Error bars represent standard deviation from the mean value. (B,C) Representative flow cytometry plots demonstrating human hematopoiesis in the bone marrow (B) and spleen (C) of an engrafted mouse.

T cell-depleted human cord blood cells containing 3×10^4^ CD34^+^ cells were transplanted into NOD-*scid IL2rγ^null^* mice as described in Materials and Methods. Approximately 3 months later, peripheral blood, spleen and bone marrow of engrafted mice were assessed for human hematopoiesis by using an antibody directed against the pan leukocyte marker human CD45 (Fig. 1). In accordance with published data [24], we obtained high levels of human hematolymphoid cell engraftment in these mice: 45–75% of cells in the lymphocyte gate (as determined by forward and light scatter) of PBMC (n = 26), 40–68% in the spleen (n = 31) and 40–60% in the bone marrow (n = 31), were hCD45^+^ suggesting that NOD-*scid IL2rγ^null^* mice support human hematopoiesis (Fig. 1A). Multilineage human hematopoiesis was assessed in the bone marrow (Fig. 1B) and spleen (Fig. 1C) of engrafted mice using antibodies against human CD19^+^ B cells, CD11c^+^ dendritic cells, CD3^+^ T cells and CD16^+^ NK cells. While the frequencies of engrafted human subsets varied between mice all the above subtypes were consistently found in engrafted mice. The data demonstrate that engrafted NOD-*scid IL2rγ^null^* mice develop all components of a functional human adaptive immune system.

### Human cells in engrafted NOD-scid IL2rγ^null^ mice support dengue infection

**Figure 2:**
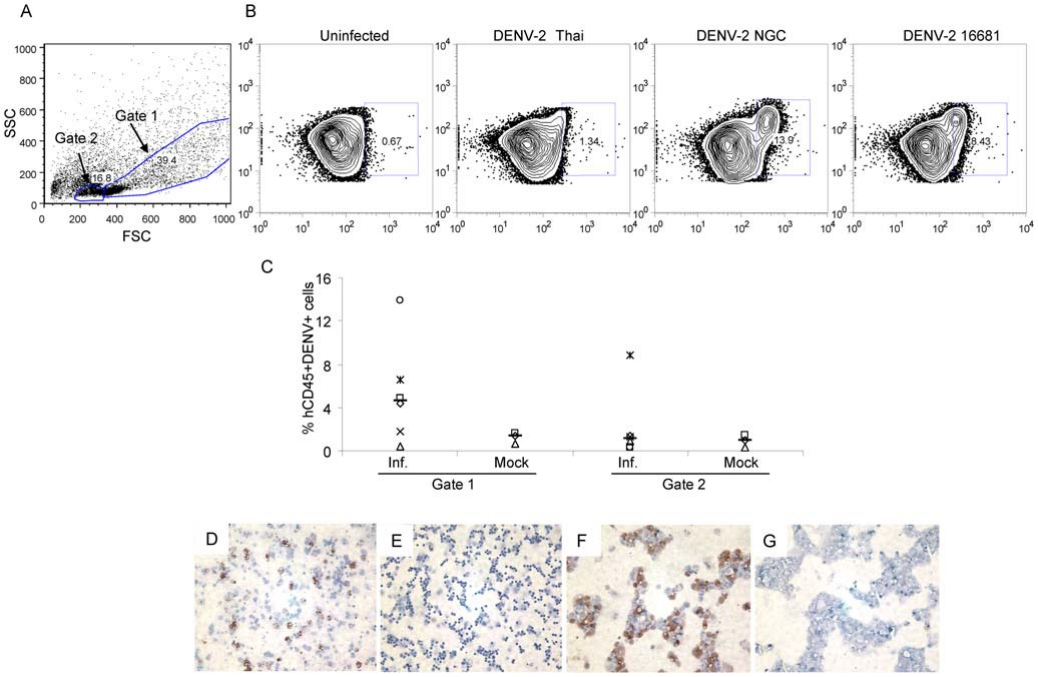
Bone marrow cells are susceptible to DENV-2 infection. Bone marrow cells (2×10^6^) from engrafted NOD-*scid IL2rγ^null^* mice were infected (m.o.i. = 2) in vitro with DENV-2 for 48 hrs. (A) Gating strategy used to identify cells with low and high side scatter. (B) Cells within the hCD45^+^ cells in gate 1 were further analyzed for viral antigen using the 3H5 mAb in uninfected and infected bone marrow cells. (C) Summary of DENV antigen expression in hCD45^+^ cells after in vitro infection with DENV-NGC. Values represent frequencies of hCD45^+^3H5^+^ cells within the respective gates. Median values are denoted by horizontal lines. Immunoperoxidase staining was performed to detect viral antigen in (D) DENV-2 infected bone marrow cells and (E) uninfected bone marrow cells using the 3H5 antibody. (F) Persistently infected Raji cells (positive control) and (G) uninfected Raji cells (negative control) were used to assess the specificity of staining.

We first wanted to determine whether human CD45^+^ cells derived from human stem cell engrafted NOD-*scid IL2rγ^null^* mice could be infected with DENV. We therefore infected bone marrow cells with clinical and laboratory strains of DENV-type 2 (DENV-2). Initial gating strategy based on forward and side scatter was used to identify cells with high side scatter (gate 1) and cells with low side scatter (gate 2) (Fig. 2A). As shown in Fig. 2B, forty-eight hrs after in vitro infection, approximately 10–15% of human CD45^+^ cells in gate 1 stained positive with the DENV-specific monoclonal antibody 3H5, following infection with DENV-2 NGC strain. In comparison, infection with a low passage Thai isolate or the DENV-2 strain 16681 yielded lower frequencies (0.5–6%) of DENV antigen-positive cells. Cells in gate 2 showed low to absent staining with 3H5 (data not shown). In subsequent experiments using bone marrows from different engrafted mice, we have detected a wide range of infectivity (0.5–15%) following in vitro infection with DENV-2 NGC (Fig. 2C).

Infected bone marrow cells derived from engrafted NOD-*scid IL2rγ^null^* mice were also assessed for dengue antigen expression by immunoperoxidase staining. We could detect DENV antigen in infected bone marrow cells (Fig. 2D) while uninfected bone marrow cells had no antigen-positive cells (Fig. 2E). The specificity of staining was confirmed using the 3H5 antibody on persistently infected Raji cells (Fig. 2F) and uninfected Raji cells (Fig. 2G). Together with the flow cytometry data, our results demonstrate that strains of DENV-2 that we tested differentially infect human cells in the bone marrow of engrafted mice.

### In vivo infection with dengue virus

Engrafted mice were next infected with 10^6^ PFU DENV-2 NGC by either the i.p. or s.c. route, the latter of which more closely mimics the natural route of infection. We monitored infected and control mice for signs of illness. Many infected mice experienced significant weight loss (greater than 25% of their initial body weight), had ruffled fur, and showed a hunched posture (data not shown). Infected non-engrafted NOD-*scid IL2rγ^null^* mice showed no signs of illness. In a small group of mice, no significant differences in platelet counts were detected in DENV-2 infected mice (921±179 10^3^/mm^3^; n = 6) compared to mock infected mice (1298±428 10^3^/mm^3^; n = 3) during acute infection (days 5–8). Visible signs of rash were detected in more than 30% of engrafted infected mice. These data indicate that several infected engrafted mice exhibited clinical features of human dengue illness although these features were not consistently observed.

### Detection of dengue virus after in vivo infection

**Figure 3:**
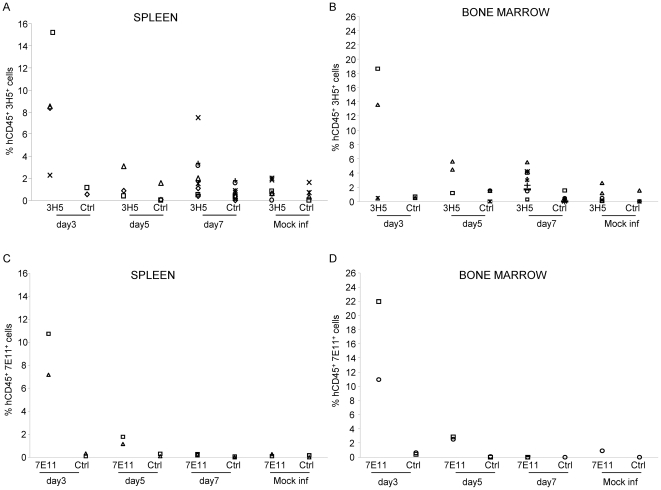
Expression of viral antigen in target tissues of mice 3–7 days after in vivo infection. Spleen and bone marrow cells were isolated from mice infected by the s.c. or i.p. route (n = 17) with DENV or mock infected (n = 5) mice 3–7 days post infection. Splenocytes and bone marrow cells were assessed for the expression of the envelope protein of DENV-2 using the zenon conjugated 3H5 mAb (A and B) or a non structural protein 1 of DENV-2 using the zenon conjugated 7E11 mAb (C and D). Data shown are frequencies of 3H5^+^, 7E11^+^ or ctrl Ab^+^ cells within the human CD45^+^ lymphocyte gate.

To determine whether viral antigen was expressed following in vivo infection, we assessed the distribution of DENV in multiple organs including the spleen and bone marrow by multiparametric flow cytometry. We utilized the DENV-2-specific mAb 3H5 directed against the envelope protein as well as the mAb 7E11 directed against the nonstructural protein NS1 of DENV-2, to detect DENV antigen in NOD-*scid IL2rγ^null^* mice 3–7 days after in vivo infection. The specificity of staining was confirmed using DENV-specific mAbs on cells from mock infected mice and control isotype antibodies on cells from infected mice (Fig. S1). Data from multiple experiments are summarized in Fig. 3. Splenocytes and bone marrow cells from mice infected 3 days prior with DENV-2 had the highest frequencies of hCD45^+^ antigen-positive cells using the 3H5 mAb with lower frequencies of hCD45^+^ antigen-positive cells detected in mice studied 5 or 7 days post infection (Fig. 3A and B). Frequencies of hCD45^+^ antigen-positive cells were similar using the 7E11 antibody and reflected the results we obtained with the 3H5 antibody in a subset of mice tested (Fig. 3C and D). Frequencies of mCD45^+^ antigen positive cells in the spleens and bone marrow of infected mice at early time points using the 3H5 antibody ranged from 0.1–1% (n = 9) which were in the range detected in mock infected mice with the exception of 1 animal which had 3.3% mCD45^+^3H5^+^ cells. Similarly, frequencies of mCD45^+^7E11^+^ cells ranged from 0–0.7% (n = 9) with frequencies in the spleens of 2 animals at 1.7 and 2.6%. Our data indicate that both structural and non-structural DENV proteins could be detected predominantly in hCD45^+^ cells following in vivo infection of engrafted mice.

### Assessment of viral loads in target organs of infected mice

**Figure 4:**
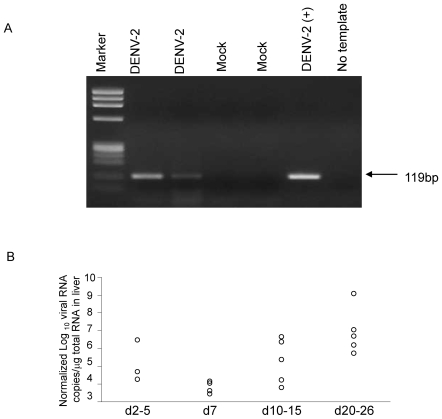
Detection of dengue viral RNA in infected engrafted NOD-*scid IL2rγ^null^* mice. (A) RNA was extracted from the livers of two infected and two mock infected mice by the s.c. route. RNA was also extracted from DENV-2 NGC and used as a positive control. Viral RNA was amplified using Lanciotti primers as described in Materials and Methods. (B) Normalized viral RNA levels on a subset of liver samples that were positive by RT-PCR.

We further assessed the presence of DENV in the target organs of infected mice. Fig. 4A demonstrates the detection of DENV RNA by RT-PCR in the livers of 2 mice 7 days after s.c. infection with DENV-2 while no RNA was detected in the livers of mock infected mice. Data from multiple experiments are summarized in Table 1. Virus was detected in greater than 50% of mice between 3 and 15 days post infection by the s.c. route. Mice infected by the i.p. route had a similar pattern of distribution throughout the body with virus being detected at early time points, although fewer mice were positive for dengue RNA by RT-PCR (Table 1). In non-engrafted NOD-*scid IL2rγ^null^* mice infected with DENV-2, no virus was detected in any of the organs at any time point (Table 1). Our data indicate that productive infection was detected only in engrafted mice. Infection by the s.c. route resulted in greater infection compared to infection by the i.p. route.

**Table 1:** Detection of DENV in organs of infected NOD-*scid IL2rγ^null^* mice.

Reconstitution	Route	Day post infection	Serum	Spleen	Bone marrow	Liver
Yes	s.c.	Day 3	n.d.**	0/2	0/2	0/2
		Day 5	1/1	3/4	1/4	2/2
		Day 7	10/13	5/8	5/8	6/8
		Day 10	0/3	0/4	1/4	3/4
		Day 13–16	14/17*	0/6	0/6	4/9*
		Day 24–26	12/12	1/1	n.d.	3/3
Yes	i.p.	Day 3	3/5	1/4	2/4	n.d.
		Day 5	n.d.	2/4	0/3	0/1
		Day 7	1/7	2/6	1/6	n.d.
		Day 10	0/1	1/2	0/2	1/1
		Day 15	n.d.	0/3	0/3	0/1
No	s.c.	Day 3	0/2	0/2	0/2	0/2
		Day 7	0/4	0/4	0/4	0/4
		Day 15	0/3	0/3	0/3	0/3

Engrafted (n>28 s.c.; n = 19 i.p.) or non-engrafted (n = 9) NOD-*scid IL2rγ^null^* mice were inoculated with 1×10^6^ PFU DENV-2 NGC strain. Mice were sacrificed at different time-points as indicated above and different organs were collected. RNA was isolated from the serum, bone marrow, spleen and liver and subjected to one-step RT-PCR as described in Materials and Methods. Values represent the number of mice that were positive by PCR in the various organs tested.

* 3 out of 17 mice and ^*^ 3 out of 9 that were positive by PCR were administered 10 µg recombinant human BLyS 3 days prior to and 3 days after infection.

** n.d.  =  not determined.

Based on a quantitative PCR assay which has detection limit of 1000 RNA copies per PCR reaction viral titers detected in the spleens of mice early after infection (days 2–7) ranged from 1.1×10^3^ to 4.3×10^5^ copies/µg RNA (n = 6 mice). In the liver, viral titers ranged from 10^4^ to 10^9^ viral genome copies/µg RNA. RNA quality was assessed by measuring copies of a housekeeping gene β-actin in liver tissue. Normalized viral RNA copies relative to β-actin levels are shown in Fig. 4B.

To confirm that the virus detected in organs of mice was due to replicating infectious virus, we passaged liver lysates from DENV-2 infected mice on C6/36 cells and detected a 5 log increase of DENV-2 genome copies in the supernatants of C6/36 cells by Q-PCR (data not shown) at 5 and 10 days post infection. These data demonstrate that infected NOD-*scid IL2rγ^null^* mice contained infectious DENV in the liver known to be a target of infection in humans.

### Antibody responses to DENV in engrafted NOD-scid IL2rγ^null^ mice

**Figure 5:**
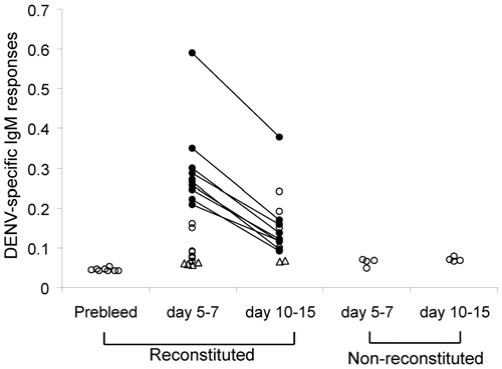
Detection of IgM-specific antibodies in the sera of infected engrafted NOD*-scid IL2rγ^null^* mice. Sera were obtained from DENV-infected engrafted (n = 8 at prebleed; n = 19 at 1 week; n = 15 at 2 weeks), non-engrafted (n = 8) and mock infected (n = 6) NOD-*scid IL2rγ^null^* mice. IgM antibodies against DENV-2 E protein were detected by ELISA. Open circles represent OD values obtained from sera of mice obtained at a single time point. Closed connected circles represent values obtained from sera of the same mice that were bled at 1 and 2 weeks after infection with DENV-2 NGC. Open triangles represent values obtained from mock infected mice. The Wilcoxin signed rank test was used to assess significance.

We assessed the generation of DENV-2-specific antibodies in the sera of infected engrafted NOD-*scid IL2rγ^null^* mice by sandwich ELISA. Significant levels of DENV-2 E-specific IgM antibodies were detected in the sera of approximately 70% (13/19) infected engrafted mice at early time points (5–7 days after infection) while 50% mice (7/15) were seropositive between days 10–15. Sera obtained from mice prior to infection, mock infected mice and infected non-engrafted mice at different time points were at background levels (Fig. 5). A cohort of mice (n = 8) was bled at 1, 2 and 3 weeks after infection with DENV-2 NGC. The highest IgM response was detected at 1 week after infection in all mice tested (Fig. 5) while DENV-specific IgM were at background values in the sera of mice obtained 3 weeks after infection (data not shown). Sera from mice with high DENV-specific IgM responses at 1 week had no responses to an unrelated protein (data not shown). We observed very low IgG responses (and only in a minority of mice studied) even at later time points (4–6 weeks). These results suggest that infected engrafted NOD-*scid IL2rγ^null^* mice are capable of generating human DENV-specific IgM antibodies.

### Human T cell responses in NOD-scid IL2rγ^null^ mice

**Figure 6:**
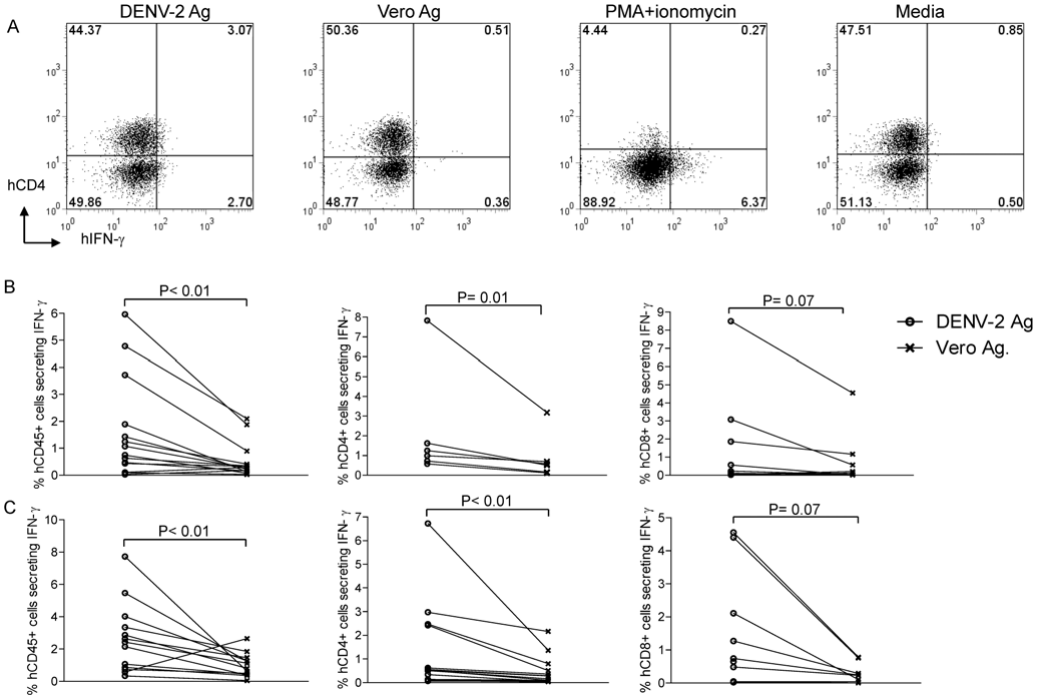
Human T cells secrete IFN-γ in response to dengue antigen stimulation. (A) Splenocytes from a mouse immunized two weeks prior with DENV-2 NGC were stimulated with an inactivated DENV-2 Ag, control vero antigen, PMA (0.1 µg/ml) + ionomycin (1 µg/ml) or media. Data shown are frequencies of human CD4^+^ T cells that produced IFN-γ in response to stimulation. (B and C) Frequencies of human CD45+, hCD4^+^ and hCD8^+^ IFN-γ secreting cells from DENV-2 infected engrafted NOD- *scid IL2rγ^null^* mice at 1 or 2 weeks post infection. The Wilcoxin signed rank test was used to assess significance.

Human T cell responses were not analyzed in humanized BALB/c-RAG2^−/−^γ_c_^−/−^ and NOD-*scid*-humanized mouse models used previously to study DENV infection [16]–[18]. We assessed DENV-specific T cell immune responses to an inactivated DENV-2 antigen used routinely in our laboratory to assess DENV-specific human CD4^+^ T cell responses by intracellular IFN-γ staining using the gating strategy shown in Fig. S2. As shown in Fig. 6A, 3.1% of human CD4^+^ cells (upper right quadrant) produced IFN-γ in response to DENV-2 antigen while 0.5% human CD4+ cells produced IFN-γ to the control vero antigen. Responses of splenocytes from the same mouse to a mitogen, PMA+ionomycin resulted in down regulation of CD4 and indicated that approximately 6% hCD45^+^ cells secreted IFN-γ.

At 1 (panel B) or 2 (panel C) weeks after infection, splenocytes from infected mice had significantly higher frequencies of human CD45^+^ cells (n = 14 mice), human CD4^+^ cells (n = 6 at week 1 and n = 14 at week 2) and human CD8 (n = 9 at week 1 and n = 10 at week 2) T cells that produced IFN-γ in response to stimulation with DENV-2 antigen compared to the control antigen (Fig. 6B and C). Splenocytes from uninfected engrafted mice had low frequencies of human CD45^+^ cells that produced IFN-γ in response to DENV-2 antigen (1.2±0.9%) or the control antigen (0.6±0.3%). These results indicate that human T cells in infected NOD-*scid IL2rγ^null^* mice were capable of eliciting DENV-specific IFN-γ responses.

### Cytokine responses to HLA A2 restricted dengue virus-specific peptides in human cord blood engrafted NOD-scid HHD Tg(HLA-A2) mice

**Figure 7:**
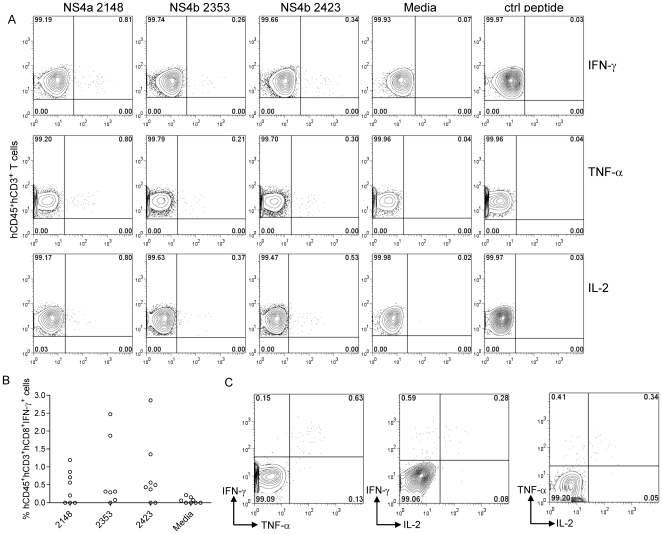
A2 restricted human T cell responses in NOD-*scid HHD Tg(HLA–A2)*mice. Splenocytes from infected NOD-*scid HHD Tg(HLA–A2)*mice were stimulated for 6 hrs with 10 µg/ml of NS4b 2353_(111–119)_, NS4b 2423_(181–189)_, and NS4a 2148_(56–64)_. A) Representative dot plots indicating the frequencies (upper right quadrant) of IFN-γ+, TNF-α^+^ and IL-2^+^ T cells by intracellular cytokine staining. B) Frequencies of hCD45^+^CD3^+^CD8^+^ IFN-γ^+^ secreting T cells in splenocytes of infected NOD-*scid IL2rγ^null^* Tg(HLA–A2/Huβ2M) and C) Representative plots demonstrating double cytokine secretion of antigen-specific T cells in response to stimulation with 10 µg/ml of NS4a 2148_(56–64)_.

In order to determine whether CD8 T cells in humanized mice are able to respond to A2 restricted virus-specific peptides previously identified in humans, we assessed IFN-γ responses in NOD*-scid IL2rγ^null^ mice* that were engrafted with HLA A2 cord blood cells. We did not detect any significant peptide-specific IFN-γ responses in splenocytes from infected NOD*-scid IL2rγ^null^ mice* to 3 HLA A2 restricted dengue-specific epitopes (data not shown). We considered the possibility that inadequate education of human T cells in the murine thymus microenvironment in these mice might result in suboptimal T cell responses. We speculated that NOD-*scid IL2rγ^null^* mice engineered to express HLA A2 might support more effective human T cell education intrathymically on human HLA A2 thus allowing for more robust human CD8 T cell responses. We developed NOD-*scid IL2rγ^null^* Tg(HLA-A2/Huβ2M) mice that express human HLA A2 on mouse cells and assessed T cell responses in mice that were infected with DENV-2. Splenocytes obtained from mice 7 days after infection with DENV-2 were stimulated with 3 HLA-A2 restricted peptides NS4b 2353_(111–119)_, NS4b 2423_(181–189)_, and NS4a 2148_(56–64)_ identified in our laboratory [25]. We detected significant frequencies of antigen-specific T cells that responded to ex vivo stimulation with all three peptides by secreting IFN-γ, TNF-α and IL-2 (Figure 7A). A summary of CD3^+^ CD8^+^ IFN-γ T cell responses obtained from splenocytes of infected mice is shown in Fig. 7B. Greater than 60% of mice had significant responses to all three peptides. The results were obtained in 3 independent cohorts of humanized mice reconstituted with cells from 3 different HLA-A2^+^ cord blood cells. Data in Fig. 7C suggest that T cell responses in immunized mice were polyfunctional since cells that secreted two cytokines (IFN-γ^+^TNF-α^+^, IFN-γ^+^IL-2^+^ and TNF-α^+^IL-2^+^) were detected in response to peptide stimulation. Together, our data indicate that humanized NOD-*scid IL2rγ^null^* Tg(HLA-A2/Huβ2M) mice are able to elicit T cell responses to known HLA A2 restricted dengue virus-specific CD8 T cell epitopes.

## Discussion

The major objective of this study was to evaluate humanized NOD-*scid IL2rγ^null^* mice as a small animal model for the study of human DENV infection. Using a low dose of a laboratory strain of DENV, our results indicate that NOD-*scid IL2rγ^null^* mice engrafted with human hematopoietic stem cells are permissive to DENV infection and generate DENV-specific human immune responses. We identified human CD45^+^ cells that were targets of DENV infection following in vitro and in vivo infection. To our knowledge, we are the first to demonstrate infection of human cells and assess the generation of DENV-specific human T cell responses in a humanized mouse model.

Our in vitro studies with clinical and laboratory passaged DENV strains demonstrate that human CD45^+^ cells in the bone marrow of engrafted NOD-*scid IL2rγ^null^* mice are differentially infected with multiple serotype-2 DENV strains. We and others have observed varying degrees of infectivity following in vitro infection of human primary cells and cell lines with clinical and laboratory passaged strains of DENV-2 [26], [27]. The vast majority of infected cells detected were human CD45^+^CD19^−^ cells and preliminary data suggest that CD11c^+^ dendritic cell precursors in the bone marrow of NOD-*scid IL2rγ^null^* mice may harbor DENV antigen (data not shown). Although some studies have identified B cells as an important target for DENV infection in humans, most data have pointed to infection of monocytes and possibly dendritic cells in vivo [15], [28]–[30]. Further studies using different serotypes and strains of DENV are required to identify predominant targets of infection after in vitro infection.

Bente and Mota et al recently reported that NOD-*scid* mice engrafted with human CD34^+^ stem cells were susceptible to infection with a low passage clinical isolate of DENV [16], [17]. Infected mice showed typical signs of dengue infection, including fever, rash, weight loss and thrombocytopenia. We did not consistently see any of these signs in our model using the laboratory passaged DENV-2 NGC, although several infected mice did show significant weight loss and had ruffled fur. We performed limited studies with the same Thai isolate of DENV-2 used by Bente et al and obtained similar results to those seen with DENV-2 NGC (data not shown). A possible explanation for differences between our studies and others may include varying strains of humanized mice used for the experiments. The NOD-*scid* mice used by Bente et al do not generate human T or B cell responses which may have influenced the ability of DENV to replicate in target tissues. Since cord blood samples from several different donors were used for reconstitution it may account for differing susceptibilities of mature human cells to DENV infection in NOD-*scid IL2rγ^null^* mice, which could contribute to the lack of consistent findings of dengue disease in our mice.

We performed most of our experiments using a single immunization with a prototype laboratory adapted strain DENV-2 NGC. The NGC strain of DENV was originally serially passaged in mouse brains and later extensively passaged in cell culture for use by many laboratories [31]. We have not detected any neurological symptoms in engrafted mice after infection with DENV-2 NGC. Additionally, no virus was detected in any organs of infected non-engrafted mice suggesting that the engrafted human cells were necessary for in vivo replication of this mouse adapted neurovirulent strain of virus and that the symptoms of dengue disease that we have observed in humanized mice is not likely due to replication in neuronal sites.

Detection of DENV RNA at multiple late time points post infection in organs known to be targets of infection in humans is encouraging and suggests that DENV-2 replicates within human cells of infected mice. Detection of the NS1 protein in predominantly hCD45^+^ cells using the mAb 7E11 suggests that the viral RNA detected by RT-PCR is due to replication of DENV within cells in humanized NOD-*scid IL2rγ^null^* mice, consistent with productive infection. Confirmation of the ability of infectious virus in liver lysates from infected mice to infect C6/36 cells further validates our PCR and flow cytometry results. Further studies will be needed to compare DENV tropism in this mouse model to those in humans.

Similar to results by Kuruvilla et al, we have detected virus in the sera (data not shown) and livers of mice that persisted for >20 days in our initial studies in a subset of NOD-*scid IL2rγ^null^* mice. Mice with high viremia lost weight and appeared sick while mice with low level or intermittent viremia had less appreciable weight loss. We are still unclear why some mice harbor DENV several weeks after infection. We speculate that the low IgG responses and inefficient human T cells generated in NOD-*scid IL2rγ^null^* mice may contribute to inefficient viral clearance in this model. In our more recent studies with NOD-*scid IL2rγ^null^* Tg(HLA-A2/Huβ2M) mice however, we have not detected any virus in the sera of mice at 2 or more weeks post infection. The results with humanized A2 transgenic NOD-*scid IL2rγ^null^* mice are highly encouraging and must be considered when evaluating varying humanized models as suitable animal models to assess dengue infection and immunity since infectious virus is cleared in approximately 2 weeks after natural infection in humans.

We detected DENV-2-specific IgM antibodies in the sera of mice 5–14 days after infection indicating that these mice are able to generate antibody responses against DENV. However, as reported by others [21], we detected little or no DENV-specific IgG (data not shown) suggesting that very low levels of class switching occurred in these mice. Variable and low IgG responses in these mice might be due to a lack of species cross-reactive cytokines in the xenogenic environment [32], [33]. We treated a small group of mice with B Lymphocyte Stimulatory Factor (BLyS), a factor known to promote human B cell survival [34], [35] and B cell engraftment in NOD-*scid IL2rγ^null^* mice (unpublished observations). Sera from BLyS-treated DENV-infected mice did not show substantially higher DENV-specific IgM or IgG responses (data not shown). Kuruvilla et al demonstrated high levels of DENV-specific IgM and IgG in stem cell engrafted RAG-hu mice several weeks post infection [18]. However, only RAG-hu mice that were immunized with a pool of four different strains of DENV-2 generated a strong antibody response while mice infected with individual strains of DENV-2 had very low levels of dengue-specific antibodies. The basis for the difference in response to the mixture of viruses was not determined. Antibody responses in our model might be enhanced by using a pool of different DENV-2 strains as done by Kuruvilla et al or by increasing the viral inoculum. However neither strategy mimics the natural course of DENV infection.

One major difference between our study and previous studies of DENV infection in humanized mouse models is our demonstration of IFN-γ production by human T cells in response to DENV-2 antigen and virus-specific peptides. Most of the mice immunized by either the i.p. or s.c. route had significant DENV-specific IFN-γ responses to the inactivated antigen with low intensity staining at 1–2 weeks post infection. Several studies have reported the important role of IFN-γ in the clearance of DENV infection [13], [36], [37]. It remains unclear how the human T cells generated in NOD-*scid IL2rγ^null^* mice are effectively educated. While mature human T cells could develop by extrathymic education and selection [24], [33], presumably most of their education occurs in the murine thymus. Melkus et al were able to overcome this problem by using NOD-*scid* mice implanted with human fetal liver and thymic tissues to measure human EBV-specific immune responses [38].

Our data using NOD-*scid IL2rγ^null^* Tg(HLA–A2/Huβ2M) mice are very encouraging and indicate that expression of human HLA molecules allow for better education of dengue-specific human T cells in humanized mice. As far as we know, we are the first to demonstrate A2 restricted peptide-specific responses in humanized mice. The ability of T cells to respond to ex vivo peptide stimulation by secreting multiple cytokines including IFN-γ, TNF-α and IL-2 demonstrate the polyfunctionality of T cells in NOD-*scid IL2rγ^null^* Tg(HLA-A2/Huβ2M) mice. The frequency of cells that respond to the 3 A2 restricted peptides by secreting IFN-γ in splenocytes of humanized mice (0.1–2.8% of total CD3^+^CD8^+^ T cells) is in line with frequencies detected in human PBMC of DENV immune donors (0.1–0.68% of total CD3^+^CD8^+^ T cells) [25]. These preliminary studies suggest that NOD-*scid IL2rγ^null^* Tg(HLA-A2/Huβ2M)mice can be used effectively to assess T cell responses to virus-specific peptides identified in humans. Further experiments will need to be performed to assess the frequency and function of dengue-specific T cells during acute infection, in memory as well as following sequential heterologous dengue virus infections.

There are still some limitations in immunodeficient *IL2rγ^null^* humanized mouse models including the requirement of human specific molecules for optimal function of the human immune system and issues of remaining innate immunity that present obstacles to human hematopoietic stem cell engraftment [39]. In addition, optimal conditions to ensure reliable human T and B cell engraftment will likely involve for example the use of neonatal rather than adult mice for engraftment and transgenic expression of select cytokines. Overcoming these limitations should be achievable in the future and will allow investigators to better recapitulate immune responses observed in immunocompetent individuals in humanized mouse models.

In summary, we have characterized a humanized mouse model with functional DENV-specific adaptive immune responses. The virologic and immunologic data presented set the stage for investigating both host and virus-specific mechanisms that control primary and sequential DENV infections. Since prior immunity is a major risk factor to developing DHF, these mice could potentially be used to study the role of cross-reactive sub-neutralizing antibodies and T cells during sequential DENV infections. Understanding the contribution of host components to severe dengue disease will lead to the development of effective therapeutics and vaccines.

## Materials and Methods

### Ethics Statement

All experiments were performed in accordance with guidelines of the Institutional Animal Care and Use Committee of the University of Massachusetts Medical School and the recommendations in the *Guide for the Care and Use of Laboratory Animals* (Institute of Laboratory Animal Resources, National Research Council, National Academy of Sciences, 1996).

### Immunodeficient Mice

NOD.*Cg-Prkdc^scid^Il2rg^tm1Wjll^/SzJ* mice (hereafter termed: NOD-*scid IL2rγ^null^*) and NOD.*Cg-Prkdc^scid^Il2rg^tm1Wjll^* Tg(HLA-A2/H2-D/β2M)1Dvs/Sz (hereafter termed NOD-*scid IL2rγ^null^* Tg(HLA-A2/Huβ2M) mice were bred at the Jackson Laboratory and subsequently maintained in the animal facilities at the University of Massachusetts Medical School [20]. The HLA-A2/Huβ2M transgene encodes a human β2-microglubulin (β2M) covalently linked to the MHC class 1, alpha1 and alpha2 binding domains of the human HLA-A2.1 gene and the alpha3, cytoplasmic and transmembrane domains of the murine H2-D^b^ [40], [41].

### Transplantation of human umbilical cord blood-derived hematopoietic stem cells in NOD-scid IL2rγ^null^ mice

Umbilical cord blood (UCB) was obtained in accordance with the Committee for the Protection of Human Subjects in Research guidelines of the University of Massachusetts Medical School. It was provided by the medical staff of the UMass Memorial Umbilical Cord Blood Donation Program. The program educates and consents mothers regarding UCB collection for research and public banking and performs collections at the time of delivery. Human umbilical cord blood (UCB) samples were depleted of red blood cells by double density Percoll gradient (1.05/1.077) and depleted of T cells using Stem Cell Technologies' (Vancouver, BC) StemSep human CD3+ depletion cocktail. CD34+ cells in the T cell-depleted UCB preparation were enumerated by flow cytometry and the sample was resuspended in phosphate buffered saline (PBS) for injection. Young adult mice 6–10 weeks of age of either sex received 240 cGy whole body irradiation (WBI) prior to intravenous delivery of 3×10^4^ CD34+ UCB in a volume of 1.0 ml via the lateral tail vein. Human cells were allowed to engraft and to generate an immune system in recipient mice for at least 12 weeks, at which time human hematolymphoid engraftment was validated by flow cytometry on peripheral blood. Successfully engrafted mice were then randomized based on engraftment for use in experiments.

### Viruses

Dengue virus serotype-2 strains New Guinea C (DENV-2 NGC), 16681 (DENV-2-16681) and a low passage DENV-2 Thai isolate were propagated in C6/36 cells cultured in RPMI 1640 (Invitrogen, Grand Island, NY) containing 5% heat-inactivated fetal calf serum (FCS; Gibco) at 28°C as previously described [42]. The low passage clinical strain was isolated from the sera of a patient on Study Day 1 (strain K0005/94, passage 2) with a diagnosis of DHF-grade 2 in a prospective study of dengue infections in Thailand [43], [44]. Virus titers were determined by plaque-forming assay on Vero cells.

### DENV infection of humanized NOD-scid IL2rγ^null^ mice

Groups of engrafted and non-engrafted NOD-*scid IL2rγ^null^* mice were inoculated s.c. or i.p. with approximately 10^6^ PFU of DENV-2. Additional groups of engrafted mice were injected with control C6/36 cell culture supernatant. Weights of mice and signs of illness were monitored every other day for 30 days. Organs (spleen, liver and bone marrow) were surgically removed from mice euthanized at different times post infection. A portion of the spleen and liver, sera and bone marrow cells were immediately frozen at −80°C for RNA analysis. The remaining portion of the spleen, bone marrow and PBMC were depleted of RBCs using RBC lysis buffer (SIGMA, St. Louis, MO) and processed to make single cell suspensions for T cell assays and to identify virus-infected cells.

### Virological Analysis by PCR

Sera, liver, bone marrow and spleen were tested for the presence of DENV RNA by RT-PCR. RNA from different organs was subjected to reverse-transcription and amplification using a Qiagen One-Step RT-PCR Kit (Qiagen) with DENV-2-specific primers D1 and TS2 as described by Lanciotti et al [45].

Viral RNA copy numbers in different organs were measured by using quantitative real-time RT-PCR based TaqMan system (Applied Biosystems, Foster City, CA) [46]. The RNA was subjected to reverse-transcription and amplification using a TaqMan One-Step RT-PCR Master Mix Reagents Kit (PE Biosystems) with DENV-2 consensus primers (forward, 5′-CAGATCTCTGATGAATAACCAACG-3′, and reverse, 5′CATTCCAAGTGAGAATCTCTTTGTCA -3′) and DENV-2 consensus TaqMan probe (6FAM-5′ATGCTGAAACGCGAGAGAAACCGC -3′-TAMRA). Probed products were quantitatively monitored by their fluorescence intensity with the ABI7300 Real-Time PCR system (PE Biosystems). DENV-2 viral RNA was used as control RNA for quantification. Viral RNA in different organs was calculated based on the standard curve of control RNA. β-actin primers were used to control for the quality of RNA (Applied Biosystem, Foster City, CA) in the liver tissue. A ratio was calculated by dividing the average Ct value obtained for all 17 samples by β-actin Ct values obtained for each sample. Ratios ranged from 0.90–1.1 for all samples indicating uniform quality of RNA in samples of liver tissue. Viral RNA copies were then normalized for RNA loading using this ratio. All assays were carried out in triplicate.

### Identification of virus-infected cells by flow cytometry

Bone marrow cells from DENV-2 infected mice were infected with different strains of DENV in vitro at an m.o.i. of 2 for 48 hrs. For in vitro and in vivo infections, cell suspensions were washed with FACS buffer (PBS with 2%FCS and 0.1% sodium azide) and blocked with CD16/CD32 (Fc block 2.4G.2) mAb for 15 min at 4°C and then stained with APC-conjugated anti-human (h) CD45 (clone 2D1) and PECy7-conjugated anti-mouse (m) CD45 (clone 30-F11) for 30 min at 4°C (BD Biosciences). Cells were washed, fixed and permeabilized for 20 min using Cytofix/CytoPerm (BD Biosciences) and stained intracellularly for the presence of DENV-2 using PE-zenon (Molecular Probes) conjugated 3H5 (DENV-2 envelope specific) or Pacific blue-zenon conjugated 7E11 (DENV-2 NS1 specific) antibodies. All cell preparations were fixed with Cytofix and analyzed on a FACSARIA flow cytometer (BD Biosciences). FlowJo (TreeStar Inc. Ashland, OR.) version 7.1 was used to analyze data.

### Identification of virus-infected cells by immunoperoxidase staining

Approximately 48 hrs after in vitro infection, 1×10^5^ cells were spun onto cytospin slides for 3 min at 600 rpm and air dried. The cells were fixed in cold methanol and then permeabilized with 0.1% TritonX-100 in PBS. Endogeneous peroxidase activity was blocked by treating cells with 3% hydrogen peroxide. The cells were further processed using the VECTASTAIN ABC Kit (VECTOR) and the DENV-2-specific monoclonal antibody 3H5 (1∶200). Color was developed by treating cells with the DAB kit (VECTOR) for 1–10 min.

### DENV isolation from organs of infected humanized NOD-scid IL2rγ^null^ mice

Frozen liver tissue was processed in small volume (1–1.5 ml) of complete media using a 70 micron cell strainer. The lysate was centrifuged at 2000 rpm for 15 min and 1 ml of the supernatant was collected. 100 µl of the supernatant from livers of 3 infected mice were used to infect a monolayer of C6/36 cells in 6 well plates for 90 min. Monolayers were next washed with media and replaced with 1.5 mls of fresh media. At different time points (1 hr, 5 days and 10 days) supernatants were collected and frozen at −80c. RNA was extracted from the C6/36 supernatant using QIAamp Viral RNA Mini Kit (Qiagen, Valencia, CA). RNA was amplified by Q-PCR as stated above.

### Detection of IFN-γ production by intracellular staining

Approximately 0.5–1×10^6^ spleen cells from DENV-infected mice were incubated with either an inactivated lysate of DENV-2 infected Vero cells (1∶40), control Vero cell lysate (1∶40), 10 µg/ml of the indicated peptide, RPMI 1640 medium (GIBCO) or phorbol myristic acid (0.1 µg/ml) + ionomycin (1 µg/ml). Vero cell lysates were prepared as previously described [42]. Golgi plug (BD Biosciences) was added to each of the above samples and incubated at 37°C. For experiments that used the inactivated dengue antigen, splenocytes were stimulated for 18 hrs while peptide stimulations were performed for 6 hrs. Cells were washed with FACS buffer, blocked with Fc block (2.4G2) for 10 min and then surface stained with FITC-hCD45 (clone 2D1), PECy7-mCD45 (clone 30-F11), Alexa-700-hCD3 (clone UCHT1), PE-hCD8 (clone H1T8a) and Pacific Blue-hCD4 (clone RPAT4) antibodies for 20 min at room temperature. Cells were washed with FACS buffer, then permeabilized using Cytofix/Cytoperm buffer (BD Biosciences) and stained with hIFN-γ (clone B27), TNF-α (clone Mab11) and IL-2 (clone MQ1-17H12) antibody for 20 min at room temperature. In some experiments the viability marker LIVE/DEAD® Aqua (Molecular Probes) was added to exclude dead cells. All cell preparations were fixed with Cytofix (BD Biosciences). Significant responses were considered to be 2 standard deviations above frequencies associated with cells stimulated with media.

### Enzyme-linked immunosorbent assay

Levels of DENV-2 envelope (E) protein-specific antibody in the serum of DENV-infected, uninfected engrafted and non-engrafted mice were determined using a standard Enzyme-linked immunosorbent assay (ELISA). 96-well microplates were coated overnight with 100 ng/well of DENV-2 E protein (Hawaii Biotech). The plates were blocked with 1% bovine serum albumin for 90 min and a 1∶20 dilution of sera diluted with PBS was added to the wells for one hour. After washing the plates, horseradish peroxidase-labeled goat anti-human IgM (Bethyl Laboratories INC. Montgomery, Texas; Catalog no. A80–100P) was added as the secondary antibody. TMB Solution (SIGMA-ALDRICH Inc., St. Louis, MO) was used as the substrate. The enzyme reaction was stopped by addition of 1M HCL and the plates were read at 450 nm. All assays were carried out in duplicate or triplicate.

### Statistical Analysis

Data are expressed as mean value ± standard deviation (SD). Significant differences between groups were determined by the Wilcoxin signed rank test. *P* values <0.05 were considered significant.

## Supporting Information

Figure S1 Detection of dengue antigen after in vivo infection. Five days after a s.c. infection with DENV-2 NGC or C6/36 supernatant, hCD45+ and mCD45+ cells from the bone marrow were assessed for dengue antigen expression. The specificity of staining was confirmed using mAbs 3H5 and 7E11 (top panel) and isotype control antibodies (middle panel) on bone marrow cells from infected mice. 3H5 and 7E11 staining on mock infected bone marrow cells (lower panel) is also shown. Values represent frequencies of antigen-positive cells within the hCD45+ or mCD45+ gate.
(1.26 MB TIF)

Figure S2 Gating strategy to identify hCD4+ and hCD8+ cells. Initial gating strategy to identify cells in the lymphocyte gate was based on forward and side scatter profiles. hCD45+ cells were next selected for using markers directed against mouse and human CD45. Viable hCD45+ were gated on by exclusion of the viability marker LIVE DEAD AQUA. T cells were next selected for by identifying CD3+ cells within the lymphocyte gate and further subsets delineated using antibodies directed against hCD4 and hCD8.
(0.74 MB TIF)
