# Supplementary figures and images for: Dengue Virus Infection and Virus-Specific HLA-A2 Restricted Immune Responses in Humanized NOD-scid IL2rγnull Mice
Source: PLoS One. 2009 Oct 5;4(10):e7251. doi: 10.1371/journal.pone.0007251 (PMC2749937; doi:10.1371/journal.pone.0007251)

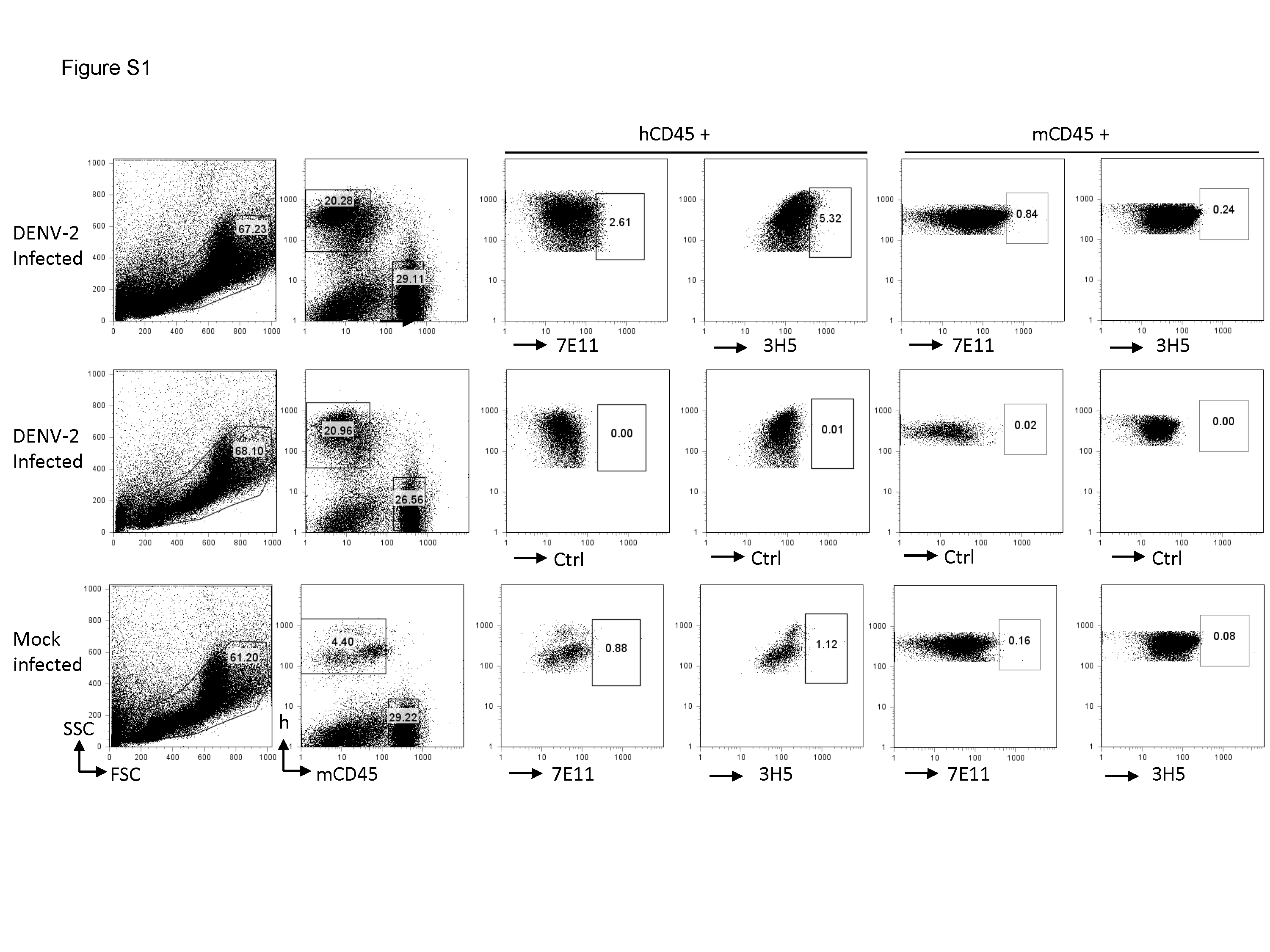

Supplement: Figure S1 — Detection of dengue antigen after in vivo infection. Five days after a s.c. infection with DENV-2 NGC or C6/36 supernatant, hCD45+ and mCD45+ cells from the bone marrow were assessed for dengue antigen expression. The specificity of staining was confirmed using mAbs 3H5 and 7E11 (top panel) and isotype control antibodies (middle panel) on bone marrow cells from infected mice. 3H5 and 7E11 staining on mock infected bone marrow cells (lower panel) is also shown. Values represent frequencies of antigen-positive cells within the hCD45+ or mCD45+ gate. (1.26 MB TIF) [file pone.0007251.s001.tif]

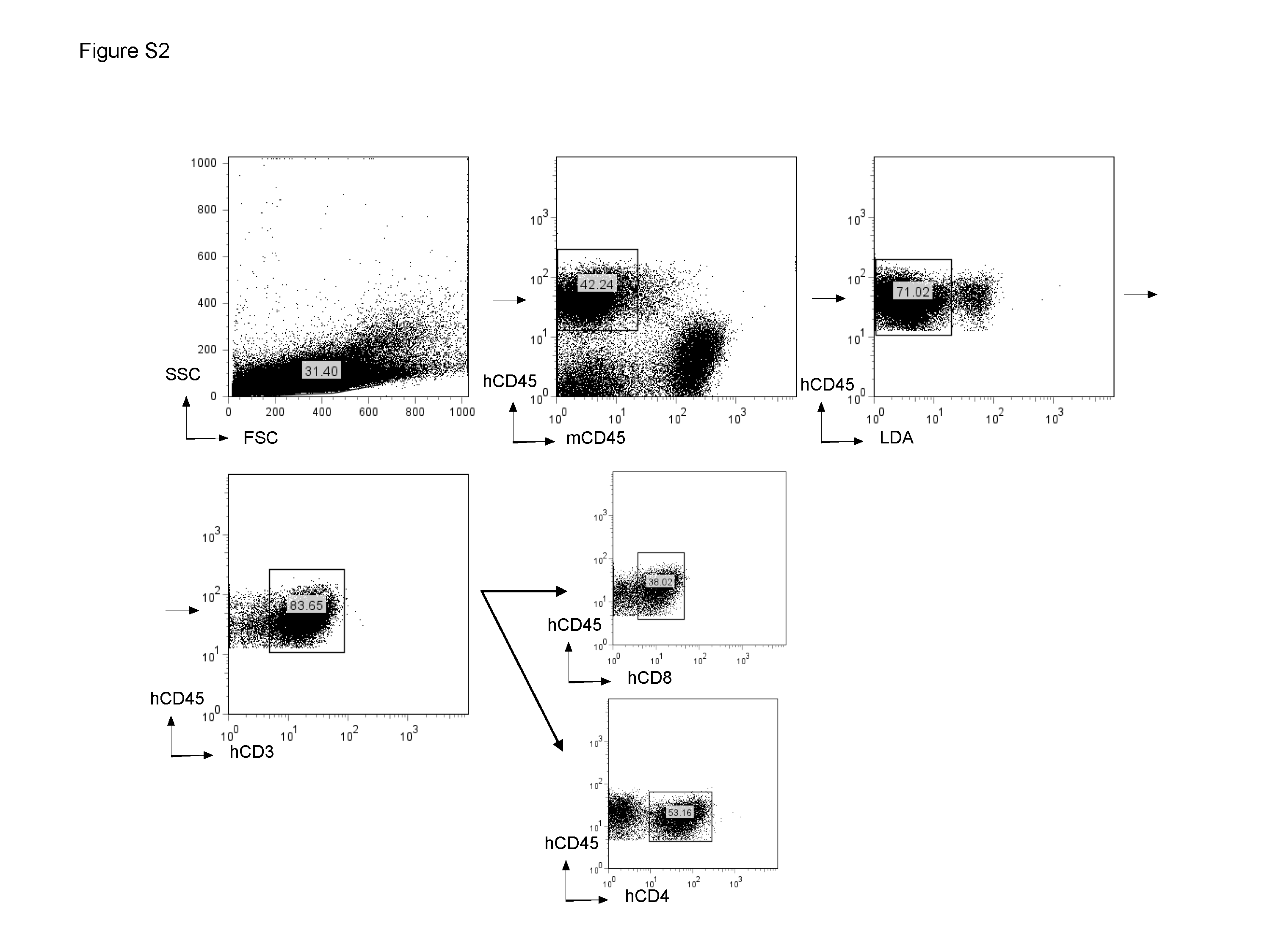

Supplement: Figure S2 — Gating strategy to identify hCD4+ and hCD8+ cells. Initial gating strategy to identify cells in the lymphocyte gate was based on forward and side scatter profiles. hCD45+ cells were next selected for using markers directed against mouse and human CD45. Viable hCD45+ were gated on by exclusion of the viability marker LIVE DEAD AQUA. T cells were next selected for by identifying CD3+ cells within the lymphocyte gate and further subsets delineated using antibodies directed against hCD4 and hCD8. (0.74 MB TIF) [file pone.0007251.s002.tif]
